# Subependymal Giant Cell Astrocytomas Without Tuberous Sclerosis: A Case Report on a Rare Medical Condition

**DOI:** 10.7759/cureus.64313

**Published:** 2024-07-11

**Authors:** Pranjali Nibe, Rupali Bavikar, Charusheela Gore, Gayatri Bhuibhar

**Affiliations:** 1 Department of Pathology, Dr. D. Y. Patil Medical College, Hospital and Research Centre, Pune, IND

**Keywords:** ttf1, young adult, brain tumors, tuberous sclerosis complex, sega

## Abstract

Subependymal giant cell astrocytomas (SEGAs) are benign, slow-growing, noninvasive tumors frequently associated with the tuberous sclerosis complex (TSC). The tumor's location and the patient's age should be considered carefully before diagnosis. Considering SEGA as a differential diagnosis, even in adult patients without TSC, is essential. In the present case, a 22-year-old male presented with a progressive headache, dizziness, and blurring of vision. Radiological investigations confirmed the site of the tumor, and a positive expression of thyroid transcription factor 1 in the ganglion cell component, along with the absence of germline mutation in TSC1 and TSC2, led to the final diagnosis of SEGA without TSC.

## Introduction

Subependymal giant cell astrocytomas (SEGAs) are slow-growing, benign, and noninvasive tumors. The World Health Organization (WHO) classifies them as grade I malignant tumors and most commonly links them to tuberous sclerosis complex (TSC) [1,2]. Of all childhood brain tumors, SEGA makes up 1.3%-1.4%. Its occurrence in relation to TSC varies from 5% to 14%; however, very few cases of SEGA without TSC have been documented [2,3].

The TSC includes subependymal nodules, retinal hamartomas, cortical nodules, and SEGAs, although very few cases of SEGA unrelated to these diseases have been reported in the literature [4]. The most common originating site of SEGA is the lateral ventricular wall, near the foramen of Monroe [5]. It most typically appears within the first two decades of life. Here, we are presenting a case of a 22-year-old male diagnosed with SEGA but without TSC based on clinical, radiological, pathological, and genetic analyses.

## Case presentation

A 22-year-old male patient presented with progressive headaches, blurred vision, and dizziness for one year. The patient was initially treated symptomatically for the presenting complaints. However, further studies were done due to no improvement in the symptoms and an increasing frequency of headache episodes.

A fundus examination was done, which revealed bilateral papilledema. General examination revealed no history of trauma, bleeding from the ear, nose, or throat, altered sensorium, or loss of consciousness. Physical examination revealed no significant neurocutaneous findings or significant past or family history.

**Figure 1:**
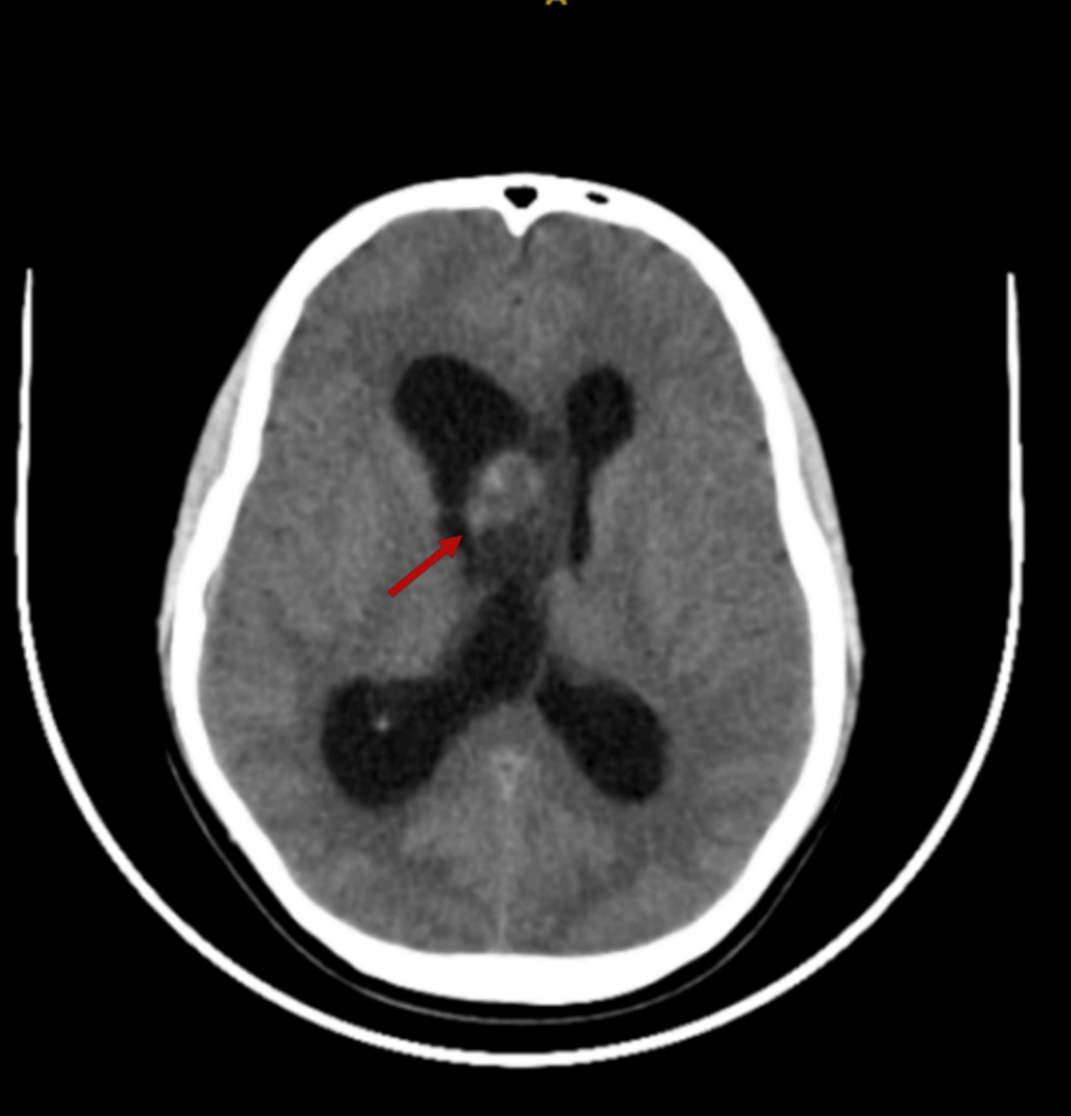
Contrast-enhanced computed tomography image, showing a well-defined heterogeneously enhancing solid cystic lesion with few nonenhancing hypodense along with few calcific foci within the foramen of Monro region (red arrow)

Computed tomography (CT) of the brain was done. It showed a well-defined, heterogeneously enhanced solid cystic lesion with few nonenhancing hypodense as well as few calcific foci within the region of foramen Monroe. This lesion was attached to the right lateral aspect of the septum pellucidum and extended into the body and anterior horn of the right lateral ventricle, measuring 27 x 26 x 25 mm (Figure 1). As a result, it was causing bilateral lateral ventricular obstructive hydrocephalus, indicating neoplastic etiology. No other findings were noted intracranially. A magnetic resonance imaging (MRI) study of the brain was performed and was consistent with the CT findings.

Based on these clinical and radiological findings, the patient underwent a neuroendoscopic excision of this space-occupying lesion (SOL) from the right lateral ventricle with septostomy and reconstruction of the foramen of Monro. Postoperative recovery was uneventful.

**Figure 2:**
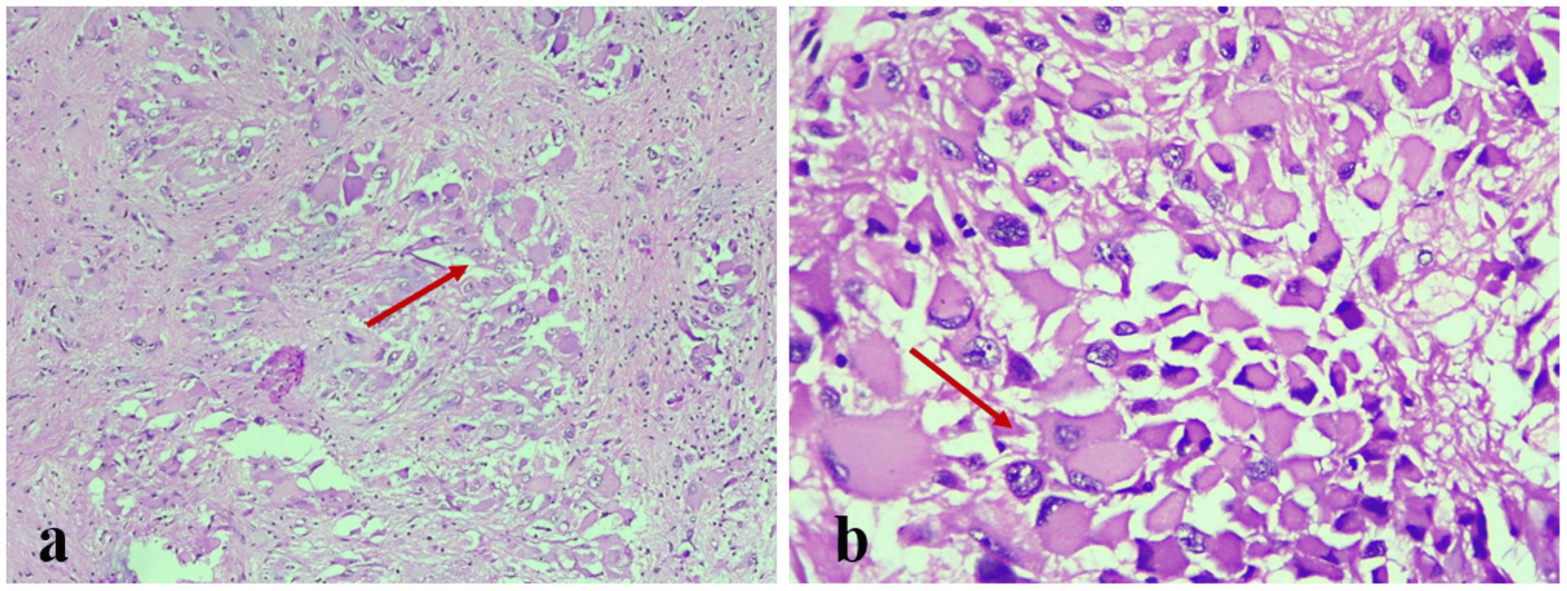
H&E-stained photomicrograph, showing clusters of round-to-polygonal-shaped ganglion cells with abundant glassy cytoplasm on (a) low-power (×100) and (b) high-power (×400) views (red arrows) H&E: hematoxylin and eosin

The removed tissue was submitted for additional histological analysis. Grossly, multiple gray-white, soft-tissue bits and pieces were received. The largest piece measured 0.7 x 0.5 x 0.2 cm, and all aggregated to 2 x 1 x 0.5 cm. All the tissue was submitted for further processing. Microscopic examination of the hematoxylin and eosin (H&E)-stained sections showed the tumor composed of neuronal and glial components. The neuronal component consists of clusters of ganglion cells, which are round to polygonal in shape, with abundant glassy cytoplasm and smaller spindle cells arranged in fascicles. In some places, perivascular rosettes are also seen (Figure 2). A glial component was noted among the ganglion cell clusters, occasionally containing Rosenthal fibers and red cells. There was no evidence of necrosis, mitosis, or perivascular lymphocytic infiltration. The two main differential diagnoses considered at this point were ganglioglioma and SEGA without tuberous sclerosis.

**Figure 3:**
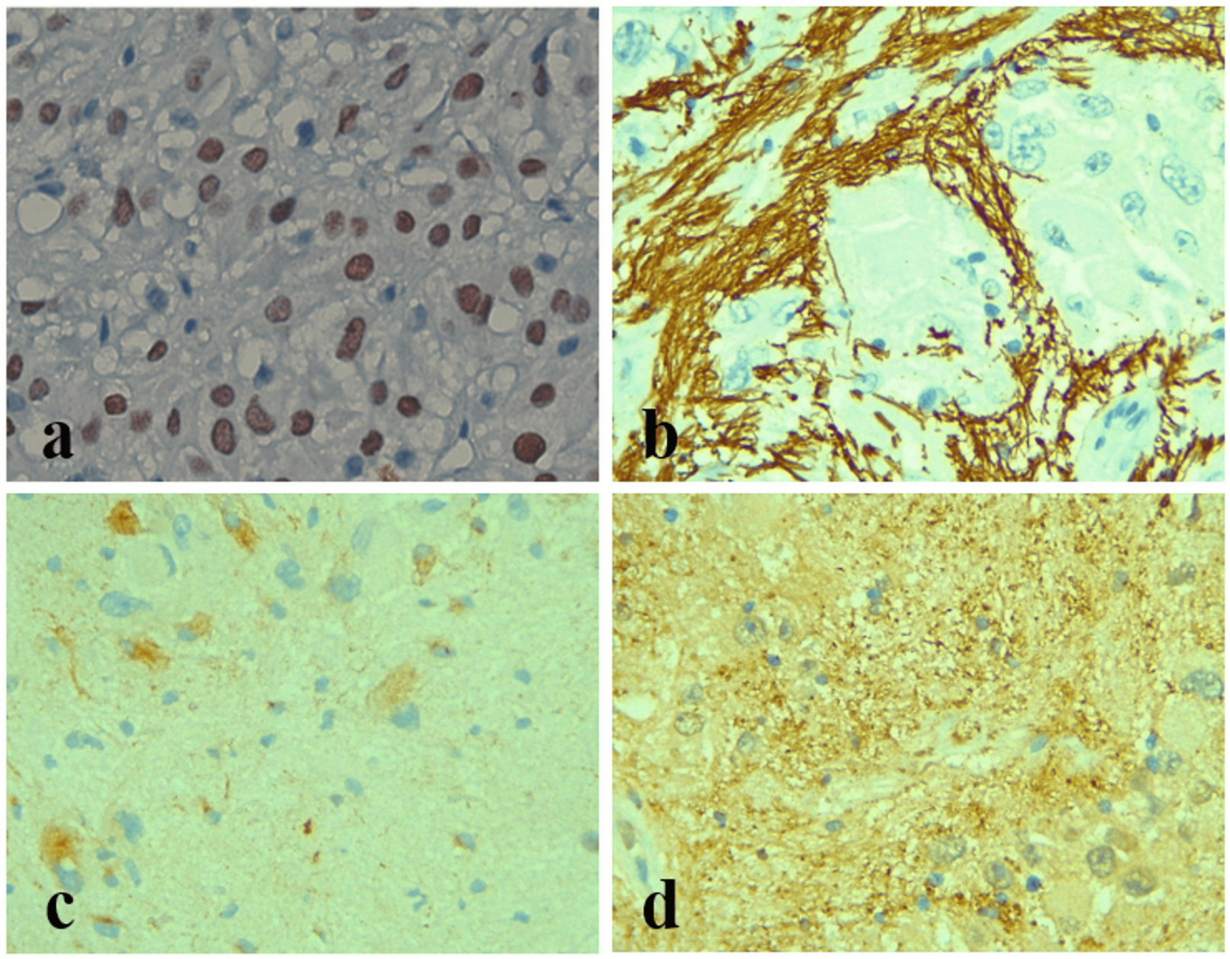
IHC findings. (a) Nuclear positivity for the TTF1 IHC marker. (b) Strong positivity of the glial component to GFAP. Focal cytoplasmic reactivity in ganglion cells to (c) synaptophysin and (d) S100 IHC: immunohistochemical; TTF1: thyroid transcription factor 1; GFAP: glial fibrillary acidic protein

Additionally, immunohistochemical (IHC) staining was performed for thyroid transcription factor 1 (TTF1), glial fibrillary acidic protein (GFAP), CD34, synaptophysin, and S100 to definitively diagnose the condition. TTF1 was found to be nuclear-positive, and the diagnosis was confirmed (Figure 3).

GFAP showed a strong reaction for the glial component, whereas S100 and synaptophysin showed a cytoplasmic reactivity in ganglion cells. Tumor cells were negative for CD34.

A study of H&E-stained slides along with IHC was pointing toward the diagnosis of SEGA. Because of the known association of SEGA with TSC, further screening for the presence of renal angiolipoma, retinal hamartomas, and oral fibromas was done to rule out any syndromic association, which revealed no significant findings. Finally, to confirm our diagnosis, further genetic testing on peripheral blood was done using next-generation sequencing methodology for both TSC1 and TSC2, which turned out to be negative. Based on these microscopic findings, IHC results, and genetic analysis results, the final diagnosis made was SEGA without TSC.

## Discussion

SEGA is characteristically observed in TSC patients. TSC is an autosomal dominant genetic disorder, and in 60% of cases, it is due to spontaneous mutation in TSC1 and TSC2 tumor suppressor genes. It is characterized by the growth of hamartomas in several organs, including the brain, heart, eyes, kidneys, lungs, and skin [4,6]. The clinical symptoms of SEGA are mainly due to its location, which is adjacent to the foramen of Monro, causing seizures and increasing intracranial pressure. In some cases, the tumor itself can obstruct the cerebrospinal fluid pathway, which can lead to hydrocephalus [3]. The common presenting symptoms are diplopia, headaches, photophobia, loss of vision, ataxia, seizures, vomiting, and detrimental effects on cognition. These symptoms are typically the result of increased intracranial pressure [6]. The cases of SEGA in association with TSC present the symptoms of tuberous sclerosis. Surgical resection has been the method used to treat this CNS tumor [2].

In a study done by Azam et al., an 11-year-old male child had been experiencing headaches and weakness in the right half of his body for 1.5 years [7]. In the current study, a 22-year-old young adult male had symptoms of progressive symptoms of headache, blurred vision, and dizziness for one year.

Reaching a definite diagnosis of SEGA needs consideration of clinical, radiological, and pathological findings. In our case, the CT and MRI investigations were nonspecific, but the location of the lesion and the age of the patient led to the preliminary differential diagnosis. It was difficult to reach the final diagnosis of SEGA because of its known strong association with TSC, which was absent in this case. The histology of SEGA can show a variety of pictures, from the traditional to the unusual, with highly pleomorphic cells. Necrosis and microvascular growth can occasionally be seen together, features that are typically seen in WHO grade IV tumors. Despite these bizarre microscopic features, the SEGA does not show aggressive behavior.

In the current study, the nuclear positivity of TTF1 (n = 1; 100%) in the ganglion cells led to the diagnosis of SEGA. In 38 cases of SEGA, Dutta et al. used clone 8G7G3/1 (1:50) for TTF-1 immunohistochemistry. According to the data, TTF-1 reactivity was observed in all 38 instances (100%) of SEGA cases [8]. Indicating the use of TTF1 in suspected SEGA cases can lead to a definitive diagnosis, resulting in patients getting an accurate treatment in time.

A revised categorization system issued in 2012 states that the diagnosis of TSC is based on clinical criteria (two major features or one major feature with two or more minor features) and genetic studies (mutation of the TSC1 or TSC2 genes) [9]. In our case, any syndromic symptoms were absent, and the TSC1 and TSC2 were negative in the germline genetic testing done on the blood sample.

Neurosurgical resection of the tumor continues to be the preferred treatment for SEGA. Total removal guarantees a conclusive recovery and prevents recurrence. Early surgical intervention has been associated with a better prognosis. In cases of worsening symptoms or progressive neurological deficits, prompt surgery is indicated [10]. The use of gamma-knife stereotactic radiosurgery remains unclear in the primary management. We continue to follow up with the patient.

## Conclusions

Considering SEGA as a differential diagnosis in cases without TSC is very important. The location of the tumor and the age of the patients should be considered carefully while reaching the diagnosis, although few cases of SEGA have been reported in the adult age group, too. It is very difficult to diagnose SEGA without TSC based on any one criterion alone; hence, clinical, radiological, and pathological correlation plays a very important role in these cases.

Since genetic testing can be very expensive and time-consuming and is not accessible everywhere, it cannot be performed in each case. In such cases, the use of the IHC marker TTF1 can play a very crucial role. Implementing the TTF1 IHC in routine practice to diagnose suspected SEGA cases, even in the absence of its syndromic association with TSC, can help reach a definite diagnosis in time. After surgical removal of this SOL, patients should be followed up for the long term to monitor the recurrence of the tumor or any symptoms.
